# Supplementary material for: Increased matrix stiffness promotes fibrogenesis of hepatic stellate cells through AP-1-induced chromatin priming
Source: Commun Biol. 2025 Jun 12;8:920. doi: 10.1038/s42003-025-08160-2 (PMC12162834; doi:10.1038/s42003-025-08160-2)

Supplemental information

Increased matrix stiffness promotes fibrogenesis of hepatic stellate cells through AP-1-induced chromatin priming

Wenxue Zhao, Weihong Yuan, Tian Dong, Wei Qi, Zhijie Feng, Cheng Li, Yujie Sun

Supplementary Figure 1

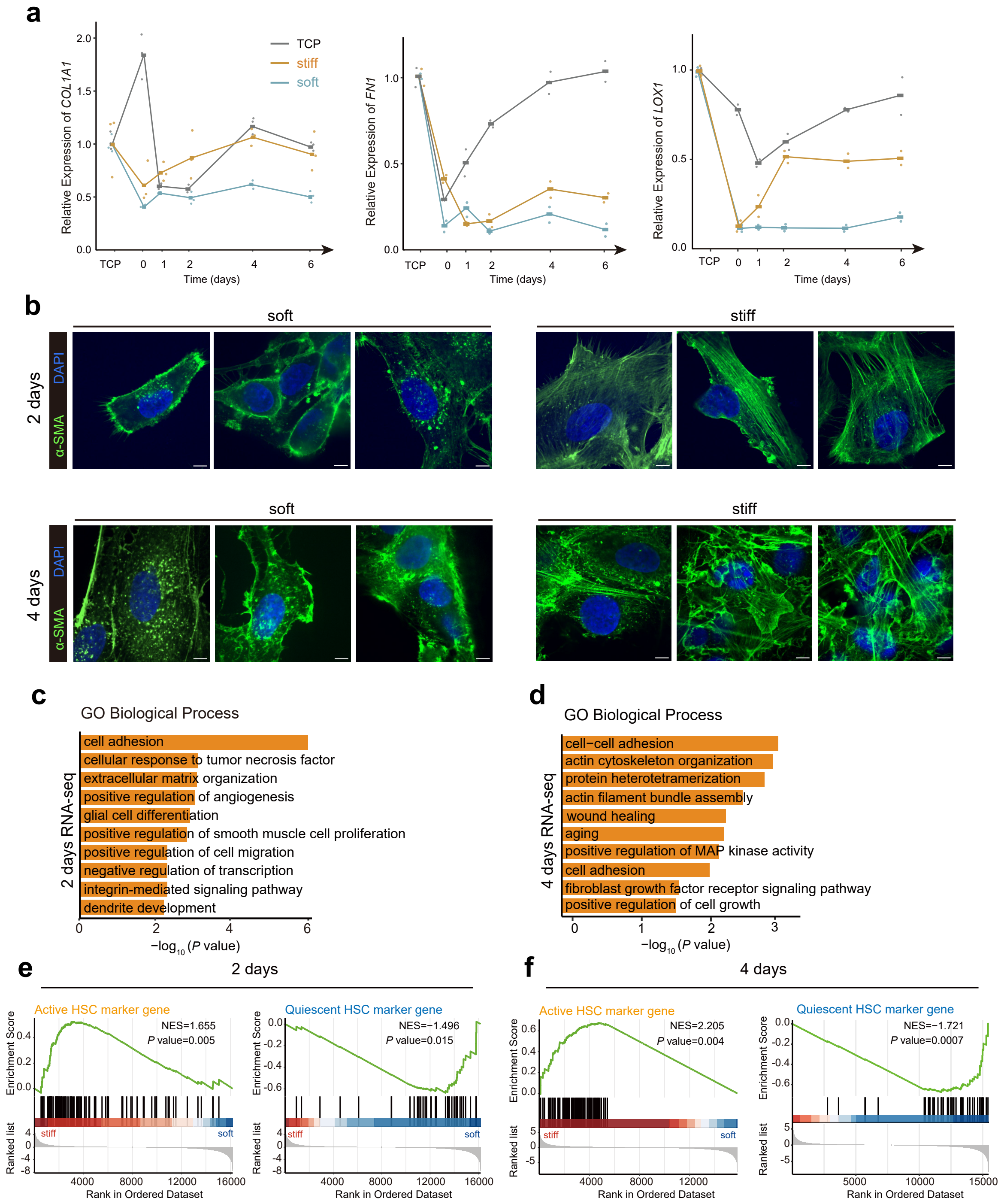

Fig S1. Different matrix stiffness facilitates HSC fibrogenesis as revealed by transcriptome profiling.

a. The relative expression levels of fibrotic genes in LX-2 cell lines over a period of days. The cells were initially plated on TCP and then isolated and reseeded onto soft and stiff matrices and TCP (used as a control). Each set of data contains at least two independent replicates. The bolded horizontal lines in each condition indicated the mean values.

b. Representative immunofluorescence images of LX-2 cells cultured on different matrices for two (up panel) or four days (bottom panel). scale bar, 10 µm.

c. GO enrichment analysis of up-regulated genes in cells cultured in the stiff matrix for two days (adjusted p value < 0.05).

d. GO enrichment analysis of up-regulated genes in cells cultured in the stiff matrix for four days (adjusted p value < 0.05).

e–f. The GSEA results show that cells cultured on soft and stiff matrices for 2 days (**e**) and 4 days (**f**) were enriched with marker genes of quiescent and active HSCs respectively.

Supplementary Figure 2

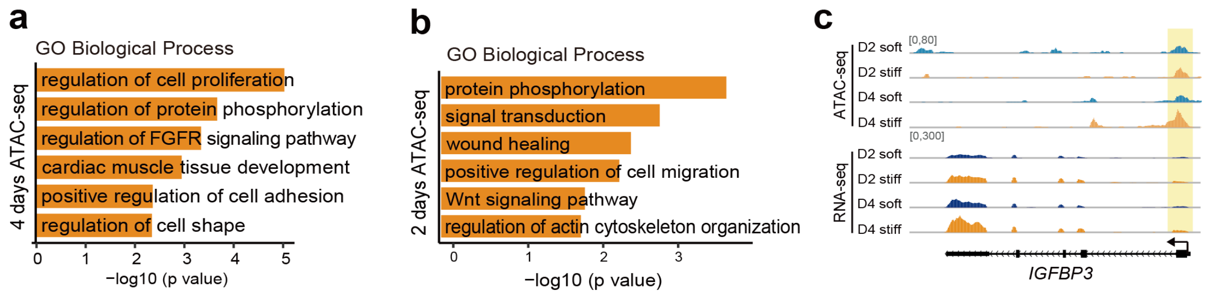

Fig S2. ATAC-seq analysis of specific chromatin accessibility clusters and adjacent gene function annotation.

a. Top enriched GO (Gene Ontology) terms for peaks (adjusted p value < 0.05) in increased accessible chromatin regions in cells cultured in the stiff matrix for four days.

b. Top enriched GO (Gene Ontology) terms for peaks (adjusted p value < 0.05) in increased accessible chromatin regions in cells cultured in stiff matrix for two days.

c. Normalized ATAC-seq signal profiles at a locus in *IGFBP3* ("D4 stiff" cluster) in cells cultured in the soft or stiff matrix, shown together with a normalized RNA-seq profile.

Supplementary Figure 3

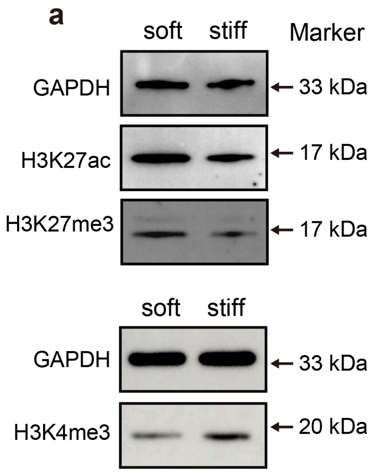

Fig. S3 Characteristics of histone modification levels in the whole genome-wide (defined in Figure 2).

a. Western blots showing the relative change of histone modification. GAPDH served as the loading control.

Supplementary Figure 4

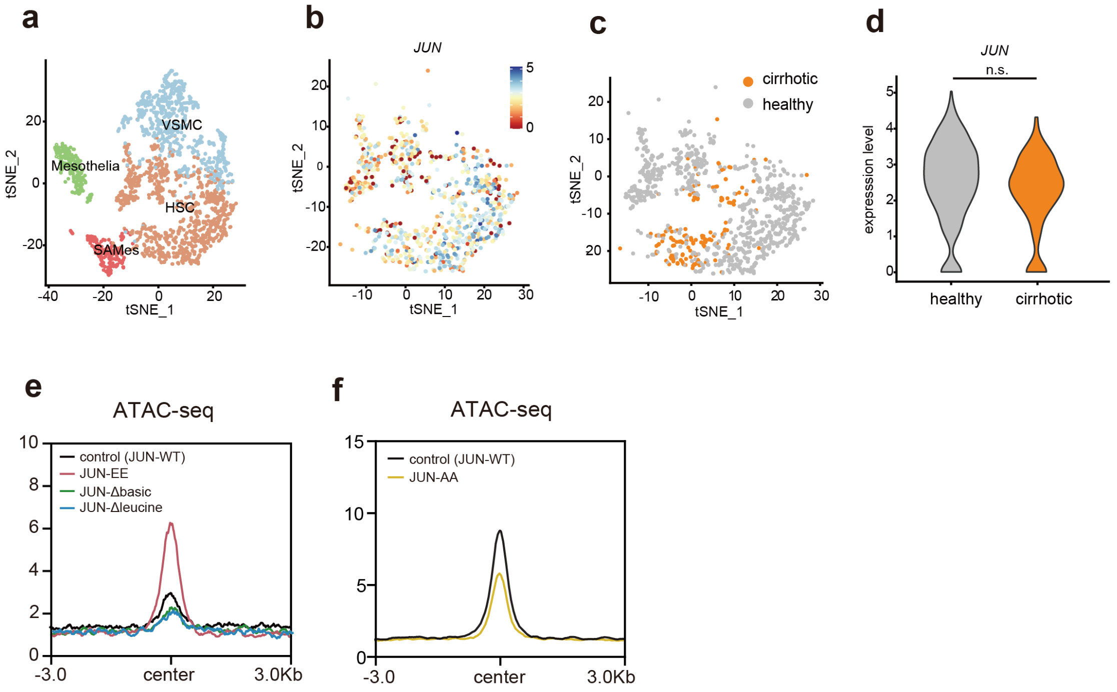

Fig. S4 p-JUN contributes to the maintenance of primed chromatin accessibility related to fibrosis.

a. Clustering of 2,031 mesenchymal cells from healthy ($n = 4$) and cirrhotic ($n = 3$) human livers identified the HSC cluster.

b. Expression of *JUN* across 946 HSCs from healthy ($n = 4$) and cirrhotic ($n = 3$) human livers (red, high; blue, low).

c. Annotation of HSC injury condition (healthy or cirrhotic).

d. Violin plot showing the expression of c-jun across HSCs from healthy ($n = 4$) and cirrhotic ($n = 3$) human livers. n.s., not significant (two-tailed wilcoxon test).

e. Normalized ATAC-seq signal profiles in the chromatin regions excluding primed chromatin following the expression of JUN-EE, JUN-EE-Δbasic and JUN-EE-Δleucine shown in **Fig. 5a**.

f. Normalized ATAC-seq signal profiles in the chromatin regions excluding primed chromatin following the expression of JUN-AA shown in **Fig. 5a**.

Supplementary Figure 5

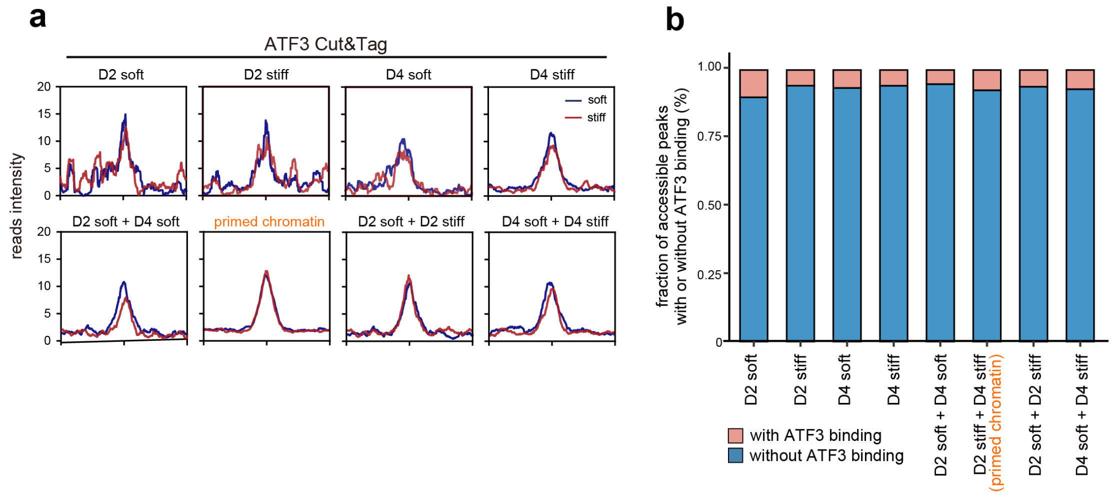

Fig. S5 Enrichment profile of ATF3 AP-1 TFs in the global and primed chromatin regions.

a. Cut&Tag signal showing the binding of ATF3 in different chromatin region clusters defined in **Fig. 2a**.

b. Stacked bar plots showing the binding faction of ATF3 in defined chromatin region clusters. Primed chromatin is highlighted in yellow.

Supplementary Figure 6

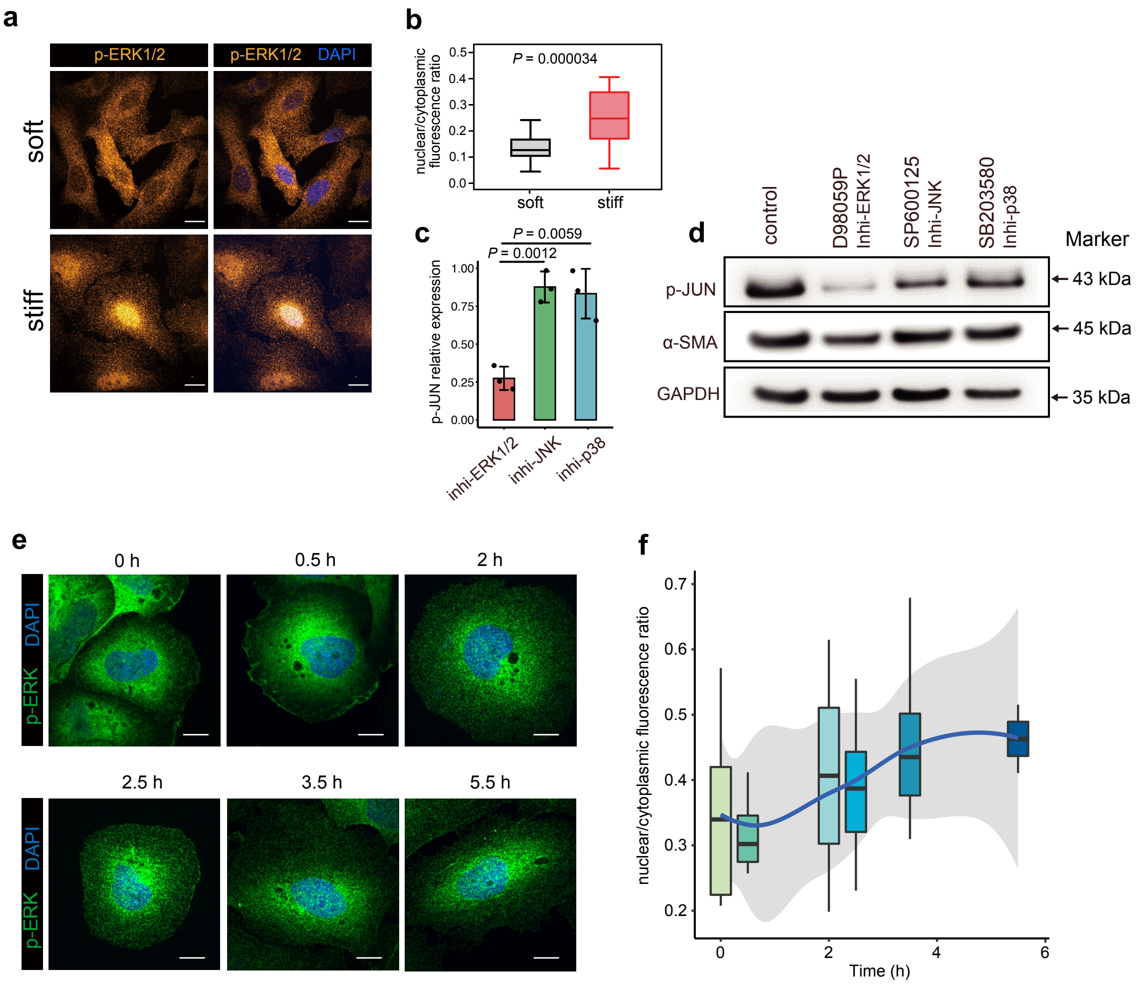

Fig. S6 Timing and duration of the nuclear ERK activity.

a. Nuclear localization of p-ERK1/2 in cells cultured on soft and stiff matrices. n=10 images per condition (scale bar, 10 μm).

b. Quantitative analysis of nuclear/cytoplasmic fluorescence ratios showing a higher p-ERK nuclear localization rate in the stiff matrix (n > 20 cells, two-tailed wilcoxon test).

c. Quantitative analysis of the western blot**,** showing the protein expression levels of p-JUN in cells cultured on the stiff matrix following inhibition of ERK, JNK, and p38 separately (one-way ANOVA; 3 independent experiments).

d. Western blots showing the protein expression levels of p-JUN in cells cultured on the stiff matrix following inhibition of ERK, JNK, and p38 separately. Western-blot are representative of three independent experiments.

e. Nuclear localization of p-ERK1/2 in cells transferred to the stiff matrixes after two-day culturation on the soft matrix (scale bar, 5 μm).

f. Quantitative analysis of nuclear/cytoplasmic fluorescence ratios showing a higher p-ERK nuclear localization rate in the stiff matrix (n > 5 cells per condition).

Supplementary Table S1

Primer	Sequence
α-SMA-F	GCCATGTTCTATCGGGTACTTC
α-SMA-R	CAGGGCTGTTTTCCCATCCAT
FN1-F	ACAACACCGAGGTGACTGAGAC
FN1-R	GGACACAACGATGCTTCCTGAG
LOX1-F	GAAACCCTTGCTCGGAAGCTGA
LOX1-R	CAGATCCAGTCTTGCGGACAAG
COL1A1-F	GAGGGCCAAGACGAAGACATC
COL1A1-R	CAGATCACGTCATCGCACAAC
COL3A1-F	GGAGCTGGCTACTTCTCGC
COL3A1-R	GGGAACATCCTCCTTCAACAG
COL8A1-F	GGGAGTGCTGCTTACCATTTC
COL8A1-R	AGCGGCTTGATCCCATAGTAG
GAPDH-F	ACCCAGAAGACTGTGGATGG
GAPDH-R	TTCAGCTCAGGGATGACCTT

Table. S1 qPCR primer sequence

The qPCR primers designed for genes of interest to detect their expression.

Uncropped blot images for Supplementary Figure 3a

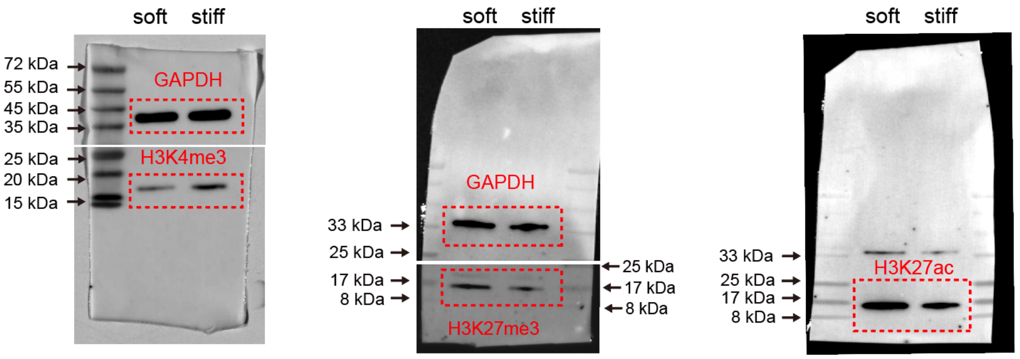

Uncropped blot images for Figure 4d

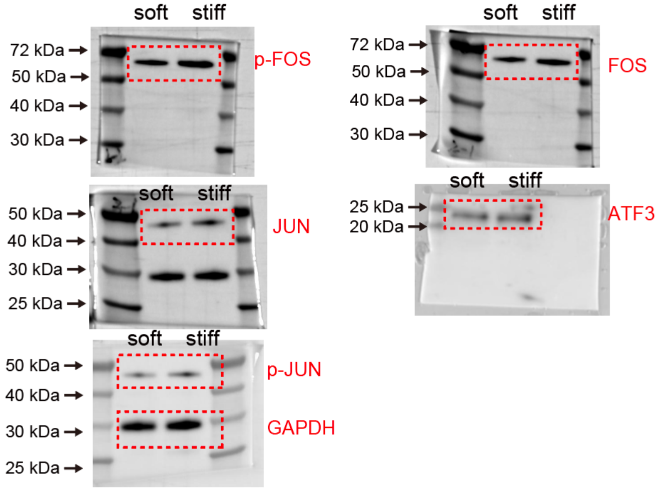

Uncropped blot images for Supplementary Figure 6d

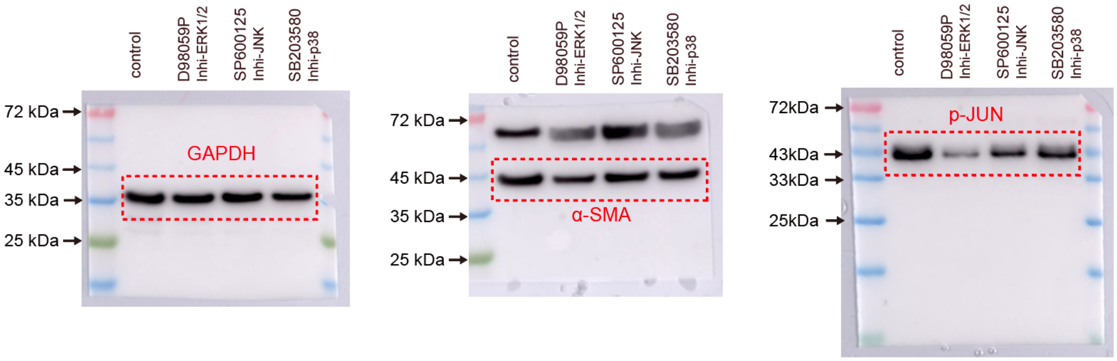

Supplement: Supplementary file 1 — Supplementary Information [file 42003_2025_8160_MOESM1_ESM.pdf]
