# Supplementary material for: Increased matrix stiffness promotes fibrogenesis of hepatic stellate cells through AP-1-induced chromatin priming
Source: Commun Biol. 2025 Jun 12;8:920. doi: 10.1038/s42003-025-08160-2 (PMC12162834; doi:10.1038/s42003-025-08160-2)
Supplement: Supplementary file 2 — Description of Additional Supplementary Materials [file 42003_2025_8160_MOESM2_ESM.pdf]

Description of Additional Supplementary Files

File name: Supplementary Data 1

Description: The numerical source data for graphs and charts
