# Supplementary material for: Increased matrix stiffness promotes fibrogenesis of hepatic stellate cells through AP-1-induced chromatin priming
Source: Commun Biol. 2025 Jun 12;8:920. doi: 10.1038/s42003-025-08160-2 (PMC12162834; doi:10.1038/s42003-025-08160-2)
Supplement: Supplementary file 4 — Reporting Summary [file 42003_2025_8160_MOESM4_ESM.pdf]

nature portfolio

Corresponding author(s): Yujie Sun, Cheng Li, Zhijie Feng

Last updated by author(s): Apr 23, 2025

Reporting Summary

Nature Portfolio wishes to improve the reproducibility of the work that we publish. This form provides structure for consistency and transparency in reporting. For further information on Nature Portfolio policies, see our Editorial Policies and the Editorial Policy Checklist.

Statistics

For all statistical analyses, confirm that the following items are present in the figure legend, table legend, main text, or Methods section.

n/a	Confirmed	
☐	☒	The exact sample size (*n*) for each experimental group/condition, given as a discrete number and unit of measurement
☐	☒	A statement on whether measurements were taken from distinct samples or whether the same sample was measured repeatedly
☐	☒	The statistical test(s) used AND whether they are one- or two-sided<br>*Only common tests should be described solely by name; describe more complex techniques in the Methods section.*
☒	☐	A description of all covariates tested
☒	☐	A description of any assumptions or corrections, such as tests of normality and adjustment for multiple comparisons
☐	☒	A full description of the statistical parameters including central tendency (e.g. means) or other basic estimates (e.g. regression coefficient) AND variation (e.g. standard deviation) or associated estimates of uncertainty (e.g. confidence intervals)
☐	☒	For null hypothesis testing, the test statistic (e.g. *F*, *t*, *r*) with confidence intervals, effect sizes, degrees of freedom and *P* value noted<br>*Give P values as exact values whenever suitable.*
☒	☐	For Bayesian analysis, information on the choice of priors and Markov chain Monte Carlo settings
☒	☐	For hierarchical and complex designs, identification of the appropriate level for tests and full reporting of outcomes
☒	☐	Estimates of effect sizes (e.g. Cohen's *d*, Pearson's *r*), indicating how they were calculated

Our web collection on statistics for biologists contains articles on many of the points above.

Software and code

Policy information about availability of computer code

Data collection	The public data sets used in our study were collected from Gene Expression Omnibus (GEO).
Data analysis	Any additional information required to reanalyze the data reported in this paper is available from the lead contact upon request. The software used in this study is described in the above section in details.

For manuscripts utilizing custom algorithms or software that are central to the research but not yet described in published literature, software must be made available to editors and reviewers. We strongly encourage code deposition in a community repository (e.g. GitHub). See the Nature Portfolio guidelines for submitting code & software for further information.

Data

Policy information about availability of data

All manuscripts must include a data availability statement. This statement should provide the following information, where applicable:

- Accession codes, unique identifiers, or web links for publicly available datasets
- A description of any restrictions on data availability
- For clinical datasets or third party data, please ensure that the statement adheres to our policy

The raw data of ATAC-seq, RNA-seq and Cut&Tag have been deposited in Gene Expression Omnibus (GEO) under the accession number GSE220703. The uncropped and unedited blot images were included in the Supplementary Information. All the raw proteomics data were deposited on Zenodo (project No.10842285). The numerical source data for graphs and charts is shown in Supplementary Data 1.

Research involving human participants, their data, or biological material

Policy information about studies with human participants or human data. See also policy information about sex, gender (identity/presentation), and sexual orientation and race, ethnicity and racism.

Reporting on sex and gender	N/A because our research used only published datasets
Reporting on race, ethnicity, or other socially relevant groupings	N/A because our research used only published datasets
Population characteristics	N/A because our research used only published datasets
Recruitment	N/A because our research used only published datasets
Ethics oversight	N/A because our research used only published datasets

Note that full information on the approval of the study protocol must also be provided in the manuscript.

Field-specific reporting

Please select the one below that is the best fit for your research. If you are not sure, read the appropriate sections before making your selection.

☒ Life sciences ☐ Behavioural & social sciences ☐ Ecological, evolutionary & environmental sciences

For a reference copy of the document with all sections, see nature.com/documents/nr-reporting-summary-flat.pdf

Life sciences study design

All studies must disclose on these points even when the disclosure is negative.

Sample size	At least three samples of each group were chosen in the biochemical experiments. At least two samples of each group were chosen in the high-throughput sequencing experiments.
Data exclusions	No data were excluded from the analyses in this study.
Replication	All attempts at replication were successful.
Randomization	Samples were allocated into experiment groups randomly.
Blinding	The investigators were not blind to group allocation during data collection and analysis. The experiments carried out were based on the specific culture conditions, thus requiring knowledge of samples to be processed.

Reporting for specific materials, systems and methods

We require information from authors about some types of materials, experimental systems and methods used in many studies. Here, indicate whether each material, system or method listed is relevant to your study. If you are not sure if a list item applies to your research, read the appropriate section before selecting a response.

Materials & experimental systems

n/a	Involved in the study	
☐	☒	Antibodies
☐	☒	Eukaryotic cell lines
☒	☐	Palaeontology and archaeology
☐	☒	Animals and other organisms
☒	☐	Clinical data
☒	☐	Dual use research of concern
☒	☐	Plants

Methods

n/a	Involved in the study	
☒	☐	ChIP-seq
☒	☐	Flow cytometry
☒	☐	MRI-based neuroimaging

Antibodies

Antibodies used	rabbit anti-SMA (Abways, CY5295)<br>rabbit anti-P-FOS (Cell Signaling Technology, 5348T)<br>rabbit anti-P-JUN (Cell Signaling Technology, 3270T)

rabbit anti-ATF3 (Abcam, ab254268)
mouse anti-c-JUN (Santa Cruz Biotechnology, sc-74543)
mouse anti-c-FOS (Santa Cruz Biotechnology, sc-166940)
mouse anti-GAPDH (Abcam, Ab8245)
rabbit anti-H3K27ac (Abcam, ab4729)
rabbit anti-H3K4me3 (Abcam, ab8580)
mouse anti-H3K27me3 (Abcam, ab6002)
Goat anti-Rabbit IgG HRP (EASYBIO, BE0101)
Goat anti-Mouse IgG HRP (EASYBIO, BE0102)
Alexa Fluor 594 donkey anti-mouse (Thermo Fisher Scientific, A21208)
Alexa Fluor 594 donkey anti-rabbit (Thermo Fisher Scientific ,A21207)
Alexa Fluor 488 donkey anti-rabbit (Thermo Fisher Scientific, A21206)
Alexa Fluor 647 goat anti-mouse (Thermo Fisher Scientific, A21235)
Alexa Fluor 488 donkey anti-mouse (Thermo Fisher Scientific, A21202)

Validation

primary antibodies:
1. abbit anti-SMA (Abways, CY5295)
website: https://www.abways.com/products.aspx?nid=3&typeid=149&key=rabbit%20anti-SMA
citation: Dou, Shi-Ding et al.,2021

2. rabbit anti-P-FOS (Cell Signaling Technology, 5348T)
website: https://www.cellsignal.com/products/primary-antibodies/phospho-c-fos-ser32-d82c12-xp-rabbit-mab/5348?srsltid=AfmBOoqYBZ9GEee_hm0iawUwiDM1iZFwANcFQ9d9L8IZgmHC8PM1YmQh
citation: Ayantika Ghosh, et. al., 2014; María Tomé, et. al., 2014

3. rabbit anti-P-JUN (Cell Signaling Technology, 3270T)
website: https://www.cellsignal.com/products/primary-antibodies/phospho-c-jun-ser73-d47g9-xp-rabbit-mab/3270
citation: Brandon Podyma, et. al., 2020; Maren Pein, et. al., 2020

4. rabbit anti-ATF3 (Abcam, ab254268)
website: https://www.abcam.com/en-us/products/primary-antibodies/atf3-antibody-epr22610-19-chip-grade-ab254268
reactivity data: 5 µg for 25 µg chromatin (Human)

5. mouse anti-c-JUN (Santa Cruz Biotechnology, sc-74543)
website: https://www.scbt.com/p/c-jun-antibody-g-4?srsltid=AfmBOoph4PnIzQ-uyYG2Vt5M17PnB7orA9eucQhC9P_fnQTU8t1Ky_H1
citation: Atak, ZK. et al. 2021. Genome Res; Li, M. et al. 2021. Cell Death Dis.

6. mouse anti-c-FOS (Santa Cruz Biotechnology, sc-166940)
website: https://www.scbt.com/zh/p/c-fos-antibody-e-8
citation: Xue, Y. et al. 2019. Nat Commun.; Prakash, N. et al. 2020. J Neurosci.

7. mouse anti-GAPDH (Abcam, Ab8245)
website: https://www.abcam.com/en-us/products/primary-antibodies/gapdh-antibody-6c5-loading-control-ab8245
citation: David W Salzman et. al., 2016. Nat Commun.; Thomas Wild et. al., 2016, Cell rep.

8. rabbit anti-H3K27ac (Abcam, ab4729)
website: https://www.abcam.com/en-us/products/primary-antibodies/histone-h3-acetyl-k27-antibody-chip-grade-ab4729
citation: Jun Wan et. al., 2017. eLife

9. rabbit anti-H3K4me3 (Abcam, ab8580)
website: https://www.abcam.com/en-us/products/primary-antibodies/histone-h3-tri-methyl-k4-antibody-chip-grade-ab8580#drawer=publications&application=chip
citation: Shan et. al., 2016

10. mouse anti-H3K27me3 (Abcam, ab6002)
website: https://www.abcam.com/en-us/products/primary-antibodies/histone-h3-tri-methyl-k27-antibody-mabcam-6002-chip-grade-ab6002
citation: Chuang et. al., 2022

Eukaryotic cell lines

Policy information about cell lines and Sex and Gender in Research

Cell line source(s)	We used the previously established human hepatic stellate cell line LX-2 (Xu et al., 2005).
Authentication	The cell lines used in the experiment were all correctly identified through short tandem repeat (STR) analysis.
Mycoplasma contamination	Cells were monthly tested for mycoplasma contamination. All cell lines tested negative for mycoplasma contamination.
Commonly misidentified lines (See ICLAC register)	No commonly misidentified cell lines were used.

Animals and other research organisms

Policy information about studies involving animals; ARRIVE guidelines recommended for reporting animal research, and Sex and Gender in Research

Laboratory animals	8-week-old C57BL/6J (MGI:3028467) male mice
Wild animals	N/A because no wild animals used in our research.
Reporting on sex	C57BL/6J male mice were used in our study. There was no sex-based analysis in the study.
Field-collected samples	N/A because no field-collected samples used in our research.
Ethics oversight	The methods were performed in accordance with relevant guidelines and regulations and approved by Institutional Animal Care and Use Committee (IACUC) of Peking University.

Note that full information on the approval of the study protocol must also be provided in the manuscript.

Plants

Seed stocks	N/A because no plants used in our research.
Novel plant genotypes	N/A because no plants used in our research.
Authentication	N/A because no plants used in our research.
